# Patient perspectives on chronic kidney disease and decision-making about treatment. Discourse of participants in the French CKD-REIN cohort study

**DOI:** 10.1007/s40620-022-01345-6

**Published:** 2022-06-13

**Authors:** Lucile Montalescot, Géraldine Dorard, Elodie Speyer, Karine Legrand, Carole Ayav, Christian Combe, Bénédicte Stengel, Aurélie Untas

**Affiliations:** 1 UNIV. NIMES, APSY-V, 30021 Nîmes Cedex 1, France; 2 Université de Paris, Laboratoire de Psychopathologie et Processus de Santé, 92100 Boulogne-Billancourt, France; 3 Université Paris-Saclay, Université Versailles Saint-Quentin-en-Yvelines, Université Paris-Sud, Inserm, Équipe Epidémiologie Clinique, CESP, 94807 Villejuif, France; 4 Clinical Epidemiology, Inserm CIC-EC, CHU de Nancy, Vandoeuvre-lès-Nancy, France; 5 Service de Néphrologie Transplantation Dialyse Aphérèses, Centre Hospitalier Universitaire de Bordeaux, and Unité INSERM U1026, Bordeaux, France

**Keywords:** Qualitative research, Treatment decision-making, Advanced CKD

## Abstract

**Background:**

Little is known about psychological issues in patients with chronic kidney disease (CKD) facing transition to kidney failure and the involvement of their family in decision-making about kidney replacement therapy (KRT). This study investigated patients’ experience of their illness, their views on KRT choice and their perception of the influence of their relatives.

**Methods:**

We conducted a qualitative study nested in the CKD-REIN prospective cohort study which included non-dialysis CKD patients from 40 nationally representative nephrology clinics. Among 1555 patients who returned a self-administered questionnaire, we used purposive sampling to select 50 participants who underwent semi-structured phone interviews with a psychologist.

**Results:**

The patients' mean age was 62.2 ± 12 years, 42% were women, and 68% had CKD stage 4–5. The analysis yielded four lexical classes: “illness rhythm”, “considering dialysis”, “family and transplantation”, and “disease, treatment choice and introspection”. When experiencing few or mild symptoms, patients tended to avoid thinking about CKD, for the prospect of dialysis was the most stressful part of their experience. Surprisingly, the importance of family appeared when they talked about transplantation decision-making, but not about choice of dialysis modality.

**Conclusions:**

Cognitive avoidance seems common in patients with advanced CKD. Transplantation and dialysis decision-making appear to be two distinct processes, with different levels of family involvement. More research is needed to better understand the frequency and impact of cognitive avoidance on patients’ well-being and decision-making.

**Graphic abstract:**

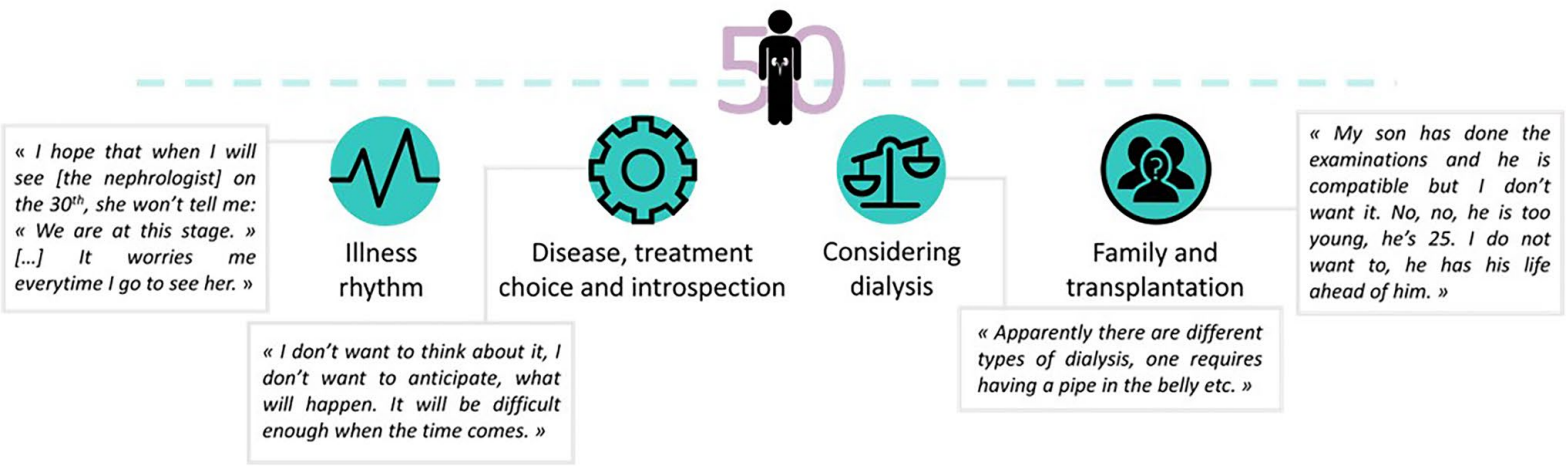

**Supplementary Information:**

The online version contains supplementary material available at 10.1007/s40620-022-01345-6.

## Introduction

Anxiety and depression are frequent in patients with moderate or advanced chronic kidney disease (CKD) [1, 2], and both are associated with worse outcomes [2, 3]. Moreover, patients reaching CKD stage 4–5 must make decisions about kidney replacement therapy (KRT), a stressful process [4]. Little is known about patients’ experience during this period. Most studies are retrospective and include patients already on dialysis, who reported feeling they lacked control over treatment choice, despite guidelines recommending shared decision-making [5, 6]. To make their choice, patients consider their preferences, and are influenced by professionals, other patients and their families [7–9]. A recent study did not find that depression and anxiety were associated with the choice of dialysis modality [10]. However, patients’ experience is likely to influence and be influenced by their mental health.

Previous studies on CKD have shown that patients often discuss treatment choices with their family [11, 12]. Qualitative studies investigating patients’ perspectives indicate that relatives provide support and information, and/or help patients accept KRT [6, 13]. Some patients consider the potential burden on their family in their choice [14]. As most of these studies are retrospective, investigating family influence on patients’ decision-making before KRT is necessary.

Statistical text analysis allows to investigate individuals’ experience through a quantitative analysis of their discourse. It investigates speech patterns (i.e., what they talk about and how they tell their experience). It aims to explore discourse through the words participants use and how these are associated with each other. It also allows to examine associations between parts of patients’ discourse and categorical variables. This method has previously been used in nephrology research [15, 16].

The aim of this study was to investigate patients’ experience of moderate and advanced CKD and their perception of KRT and the influence of their relatives.

## Methods

### Design and setting

The CKD-Renal Epidemiology and Information Network (CKD-REIN) study is a prospective cohort which enrolled 3033 patients with non-dialysis CKD from 40 nationally representative nephrology clinics between 2013 and 2016. This work was conducted under the approval of relevant ethics committees (including CCTIRS, N°12.360). Data were collected annually, including self-administered questionnaires. The study protocol and patient baseline characteristics have previously been published [17, 18]. At the third-year follow-up (2016–2019), 2260 patients who had not initiated KRT were invited to participate in a qualitative investigation by phone interview with a psychologist. Among the 1,555 pre-KRT patients who returned the self-administered questionnaire, 719 agreed to take part in the interview. We then used purposive sampling to select 50 participants so as to ensure diversity in our sample with regard to age, gender and CKD stage. The goal of this type of sampling is to intentionally select participants so they represent some explicit predefined traits. This provides for relatively equal numbers of different categories to enable exploration of the lived experience of each of these groups [19].

### Data collection

Clinical research associates collected clinical data from medical records. The CKD-EPI equation was used to estimate glomerular filtration rates (GFR). Participants completed a questionnaire collecting sociodemographic information and several patient-reported measurements. The Hospital Anxiety and Depression Scale was used with possible scores ranging from 0 to 21 [20, 21]. A score above 8 was used to identify patients with significant anxiety symptoms.

The Center for Epidemiologic Studies-Depression scale was used to screen for depression symptoms [22]. Psychometric properties were explored among the patients participating in the CKDREIN-*Famille* study. Two items with poor saturation were excluded. A final eight-item version, with possible scores ranging from 0 to 24, showed satisfactory internal consistency (Cronbach’s *α* = 0.82) and fit (Comparative Fit Index = 0.98, Goodness-of-Fit Index = 0.98, Standardized Root Mean Square Residual = 0.027). A score above 8 was used to identify patients with significant depression symptoms. This cut-off was determined with a cross-multiplication based on the initial version threshold. A first interview guide was pilot tested with 3 patients. Then, qualitative data were collected by LM from audio-recorded semi-structured individual phone interviews (see interview guide in Box 1). She was a PhD student and received training from AU regarding qualitative research. She did not know the participants beforehand. She introduced herself as a psychologist-researcher then presented the interview as a means to know more about patients’ experience of CKD. Participants were asked to be in a room alone for the interview. The interviews, which took place between January 2018 and January 2019, lasted a median of 42 min [range 16–95]. LM took notes during the interviews to help her prompt the participants, however no field notes were made after the exchanges. All data were transcribed verbatim and included in the analysis. A short subjectivity statement and a description of the interpretation process are available as supplementary material. We used the Consolidated Criteria for Reporting Qualitative Research (COREQ) [23] to report key aspects of our study.

Box 1: Topics addressed in the interview guide

- Current experience with CKD
- Knowledge and perception of KRT
- Discussions of KRT with healthcare professionals
- Discussions with family and friends and factors affecting treatment choice
- Roles in decision-making

### Analysis

We described the patients’ characteristics at the time of the interview and compared them between patients who consented to be interviewed and those who did not. All interviews were transcribed. We used ALCESTE^®^ software (Analysis of Co-occurring Lexemes in a Set of Text Segments) to perform quantitative analyses of qualitative data based on the units of contexts, i.e., elements of the interviews roughly equivalent to sentences used by individuals [24]. It allows for an inductive analysis of the data. Although ALCESTE^®^ software was developed in France it is available and used in several other languages. ALCESTE^®^ performs a descending hierarchical classification (DHC) which yields classes of words, to which the researcher then gives meaning by investigating the words comprised in each class and their associations (see supplementary material regarding the interpretation process). The association of each word with each class is tested by a Chi-square test. A detailed description of the ALCESTE® process is available as a supplementary file. This type of analysis was chosen because it allows a quantitative analysis of large text data while keeping the strength of a qualitative approach. The software provides an automatic analysis of speech, which forms classes without the subjectivity of the researcher [15]. Moreover, ALCESTE^®^ emphasizes not only *what* participants talk about but also *how* they tell their experience, allowing to identify patients’ implicit views.

Data saturation is a methodological principle in qualitative research referring to the point in the analytic process when no new information is discovered in the analysis and data become redundant [25]. Data saturation often occurs after 12 interviews [26]. As stated above, we chose to undertake 50 to ensure diversity. LM analyzed ALCESTE^®^ outputs supervised by AU. The details of her interpretation process are available in the supplementary material.

A chi-square test was also performed to assess the strength of the association between patient characteristics and the classes. The following categorical variables were considered in this analysis: CKD stage, participation in patient education sessions about KRT in the past year (yes/no), discussion of treatment choices within the family (yes/no), anxiety (yes/no), and depression (yes/no).

## Results

### Patients’ characteristics

Patients who consented to be interviewed were younger, were less likely to be widowed, had a higher education level, greater anxiety, had better literacy skills and had more often discussed KRT with relatives than those who did not consent to an interview (see supplementary material). Interviewed patients’ mean age was 62.2 (± 12.2), 42% were women and 68.0% had stage 4–5 CKD (Table 1).

**Table 1:** Characteristics of the sample cohort participating in the interview

Variables	Patients who took part in an interview
	*N* = 50
Sociodemographics	
Age (mean (SD))	62.2 (12.2)
Gender (% women)	42.0%
GFR (mean (SD))	24.5 (11.6)
CKD stage at 3-year follow-up	
Stage 2–3	28.0%
Stage 4–5	68.0%
NA	4.0%
Attended patient education on KRT (% yes)	26.0%
NA	8.0%
Marital status	
Single	16.0%
Divorced	8.0%
Married	66.0%
Widowed	8.0%
NA	2.0%
Lives alone	22.0%
Occupational situation	
Retired	50.0%
Full-time job	28.0%
Part-time job	6.0%
Unemployed	4.0%
Disability leave	10.0%
Education level	
≤ 9	2.0%
10–12	46.0%
> 12	50.0%
Good literacy skills^a^	84%
Depression^b^	
Mean (SD) (HADS score ≥ 8)	7.4 (5.0)
Depressed (CES-D score ≥ 8)	38.0%
NA	10.0%
Anxiety^c^	
Mean (SD)	5.3 (3.3)
Anxious	24.0%
NA	0.0%
Discussion with family members	78.0%
NA	0.0%

*GFR* for glomerular filtration rate; NA for Missing Data; KRT for Kidney Replacement Therapy

^a^Patients were considered as having good health literacy skills when they reported they never needed help reading documents written by health professionals

^b^Measured by the Center for Epidemiological Studies Depression Scale (Kohout et al. [22])

^c^Measured by the Hospital Anxiety and Depression Scale (Zigmond and Snaith [20])

### Results of the lexicometric analysis

**Fig. 1:**
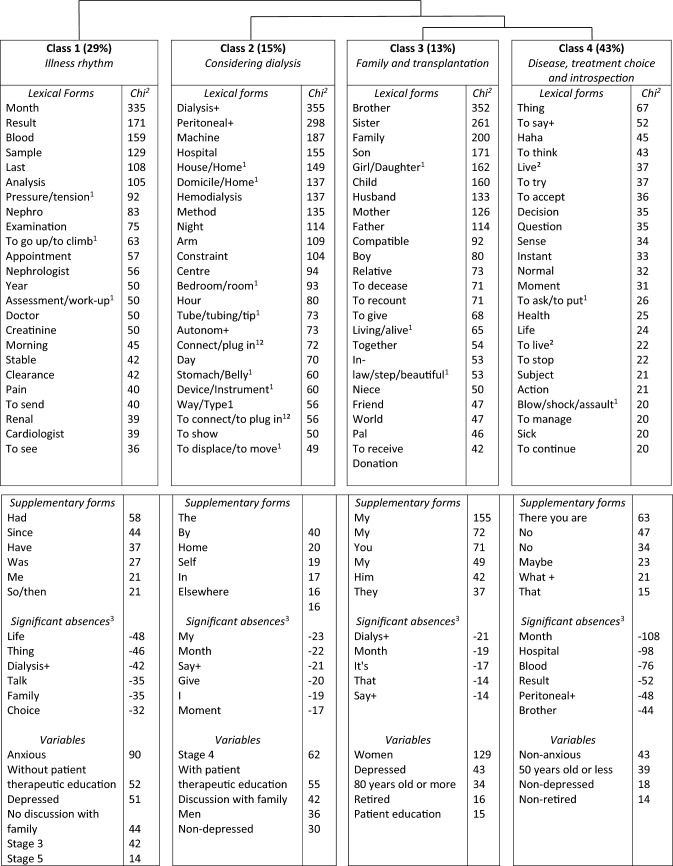
Figure of the descendant hierarchical classification. ^1^These occurrences have different meanings in French. ²Some conjugated forms of these verbs were analyzed separately from their lemmatized form because they can have homonyms.^3^As a chi-square cannot be negative, when a minus precedes the Chi-square value it is used by the software to indicate a “relative absence” of the word in the class

The corpus comprising all the interviews contained 264,875 different lexical forms (i.e., words) and 5715 units of context. The DHC shows the lexical forms and the supplementary forms associated with each class and how the classes are linked with each other (Fig. 1). ALCESTE^®^ classified 68% of the corpus into four classes.

#### Class 1: rhythm of the illness

The first class encompasses 29% of the classified corpus**,** it is characterized by temporal indicators, e.g., “week” and “year”, and words showing temporal relationships, e.g., “since” and “when”. It is divided into two subclasses.

The “CKD monitoring” subclass is composed of words such as “month” and month names. These terms co-occur with words pertaining to monitoring such as “result”, “appointment”, “blood” and “test/taken/sample”. CKD manifests itself through these medical events and their repetition (Table 2, Class 1, Excerpt 1).

**Table 2:** Excerpts from the analysis

Class 1: illness rhythm	CKD monitoring	Excerpt 1	“I hope that on the 30th, when I go see her, she doesn't say to me 'We're at that stage'! To say to you, uh, so yeah. Um. Bah, she makes me anxious each time I go see her. Each time I'm waiting for a result, each time I go see her.” Tony, age 54
	Managing an illness among others	Excerpt 2	“The arthritis in my hands worries me more than my kidney insufficiency! Yes! Because … < coughing sound > at the end of the day, at the end of the day, pain comes.” Guillaume, age 55
		Excerpt 3	“I had a blood sample and then I saw my general practitioner the day or two before … seeing the nephrologist. And in the results that, that there were, there were numbers that were very very bad. And that was really a blow to my morale. And then two days later, I saw the nephrologist. I talked to her about it. She said to me, 'But that, that's a rate that's part of the analysis but we never do anything about it!' “ Samuel, age 54
		Excerpt 4	“I go see the cardiologist, I go see the diabetes specialist, and I go see the nephrologist.” Monique, age 82
Class 2: considering dialysis	Dialysis modalities and day-to-day life	Excerpt 1	“Uh I know that there are people who do dialysis at home, uh, who spend the night with a machine that uh do the session/purification purifies the blood of its, its toxins.” Joël, age 82
		Excerpt 2	“Peritoneal dialysis at one's house, eh, well, at home, uh I think that it avoids spending hours in the hospital, so to be able to stay home, but under what conditions?” André, age 65
		Excerpt 3	“I don't know, I'd like to opt for dialysis at, at night. So yeah, it's that.” Adèle, age 69
	Patient education and treatment choice	Excerpt 4	“I am pretty much aware of everything and, not all of course, I'm pretty much up to date, so yeah. Well, uh, good, he explained to me first, how things were going and he sent me to take some classes, and in those courses they taught me the different systems of dialysis and the possibilities I had.” Dimitri, age 69
		Excerpt 5	“I don't know how it goes, but it must certainly be very aseptic, anyway well, both systems have their advantages and their disadvantages. I know, I think, anyway, that the system with dialysis in the arm, uses up the veins more, according to what I've read.” Pedro, age 56
		Excerpt 6	“You have to go to the hospital, I don't know anymore if it was 2, 3, or 4 times a week! With some, there! It's a huge constraint! Huge huge constraint!” Samuel, age 54
Class 3: family and transplantation	Talking about the illness	Excerpt 1	“I don’t tell my mother all of it, to not bother her too much with it. Uh, and then you have to have some privacy, so I am not always going to recount what I have, what's well, what's not well.” Jérôme, age 61
		Excerpt 2	“Everyone turned their back on me. So uh well I don't talk about it. No, no I don't see anyone anymore.” Judith, age 61
		Excerpt 3	“But otherwise, with the family, my nephews, all of that, everyone is, I have cousins who know, my sister-in-law, etc.” No, no among us, it’s ok, we talk about it easily. But not with Granny” Anouk, age 66
	Talking about transplantation	Excerpt 4	“[About a transplant from a living donor or a deceased donor] I have no brother or sister, mom is a little too old. 80 years old, she's my mother so, good, an 80-year old kidney, knowing that she's sick… So a deceased donor, yes.” Paul, age 61
		Excerpt 5	“My son did them [the predonation examinations] and he's compatible but I don't want to. No. No, he's too young, he's 25, I don't want it, he has his whole life in front of him.” Nicole, age 52
		Excerpt 6	“With my husband … we talked about it. I said to him: 'You know, it would be good …' and all, and 'ooh la la' he said to me, 'but wait', he says to me 'to give a kidney but you realize, after uh', so it's these things you see, you talk a little in a vacuum, you don't really know because uh he tells me 'but you know that if I give you one of my kidneys, if of course, I'm compatible, if I give you one my kidneys,' he says to me, “to live with only one kidney, it's uh, it must not be easy.'“ Christine, age 61
		Excerpt 7	“It's not more complicated. It's something else. First, someone has to die, and they have to be compatible. And well, I have no desire for someone to die to save my life.” Ezra, age 60
Class 4: disease, treatment choice and introspection	A “normal” life	Excerpt 1	I don't feel sick, I, for me, I'm not sick, in fact.” Audrey, age 65
		Excerpt 2	“Pff, what I'm living < dealing with > badly, it's the fatigue. The impression of being, uh, passive. Anouk, age 66
		Excerpt 3	“That doesn't bother me in my life! I find myself, in quotes, uh “normal.” Céline, age 50
		Excerpt 4	“I don't know. No one knows, and no one is able to say really how it is going to develop! Especially, to say to yourself for a moment, uh, bah, all the uncertainties around a transplant, since I, I myself would like to avoid dialysis at any cost!” Cassandre, age 46
		Excerpt 5	“Pff, well, I don’t want to live like him. But I’m way younger than him, my dad died when he was 80, 81 uh pff. I have time to see this coming. Haha.” Abby, age 61
	Avoiding thinking about CKD	Excerpt 6	“I try not to think about it, I don't want to anticipate what's going to happen. It will already be, uh, yeah. Uh complicated, when the time comes. Céline, age 50
		Excerpt 7	“I don't think about it. No. Bah, it's a little like a sword of Damocles, so, and I say to myself, when is it going to fall?” Cassandre, age 46
		Excerpt 8	“Still, it's one of those things you think about, anyway, I'm not going to tell you that I'm completely uh ignorant of all those questions and that I totally never think about those questions.” Ginette, age 73
		Excerpt 9	“From the perspective of a good state of mind, I try to be careful about a lot of things, my meals, stuff like that.” Ginette, age 73
		Excerpt 10	“I know that it's going to be very complicated for me to manage. Um, uh I, my morale is going to be very beaten down.” Céline, age 50
	Treatment choice and acceptation facing research participation	Excerpt 11	“If it continues like this, it's fine. I sleep well, I eat well, I walk in the woods a lot.” Colin, age 80
		Excerpt 12	“I have trouble accepting that uh that one day or another I might have to start dialysis! Céline, age 50
		Excerpt 13	“It's not easy to answer this question because it's true that I realize, well I realize, yes, that I haven't asked myself them really.” Michel, age 70

CKD does not hurt, even though “pain” belongs to the “Managing an illness among others” subclass. This word appears in negative sentences or refers to other diseases (Class 1, Excerpt 2). Moreover, this subclass is composed of words designating medical fields: “cardiologist” and “urologist”. This result shows that CKD is one illness among others (Class 1, Excerpts 3–4).

Words pertaining to KRT and decision-making, e.g., “choice” or “dialysis”, are significantly absent in class 1 and suggest that before they face kidney failure, patients are not thinking about KRT. Moreover, the absence of the terminology of family from this class indicates that CKD monitoring is an individual experience.

This class is associated with several variables: anxious and depressed patients, non-attendance at patient education about KRT, and not talking about KRT with relatives. Patients with stages 3 and 5 CKD are associated with this class while those living with stage 4 CKD are significantly absent from it.

#### Class 2: considering dialysis

This class covers 15% of the classified corpus and is divided into two subclasses.

“Dialys + ”, “machine”, “peritoneal + ” and “house/home” appear in the “Dialysis modalities and daily life” subclass. Patients describe different types of dialysis and how these could be implemented in their life. “Know”, “sort/kind”, “near” (found in the expression “à peu près”, literally, “a little near”, or roughly/almost) and “uh” show how patients may perceive their knowledge on this subject as approximate (Class 2, Excerpt 1).

Dialysis modalities are described according to where they can be implemented (“house/home”, “hospital”, “at”), when and how long the treatment takes place (“hour”, “night”) and the degree of freedom allowed (“autonom + ”, “free”) (Class 2, Excerpt 2). “Opt” and “choose” show the beginning of a decision-making process (Class 2, Excerpt 3).

The “Patient education and treatment choice” subclass includes words related to patient education (e.g., “meeting”, “to show” and “information”) (Class 2, Excerpt 4) which allows patients to grasp how treatments “function”, obtain information and understand the “advantages”/ “disadvantages” of each modality (Class 2, Excerpt 5). “Have to”, “can/be able”, “etc.” and “constraint” show that the obligations of dialysis are perceived as numerous and burdensome (Class 2, Excerpt 6). Words referring to family are significantly absent from class 2, relatives may not be considered in reflections on dialysis modalities.

This class is associated with: stage 4 CKD, attendance at patient education sessions, discussions about treatment with their family, and no depression.

#### Class 3: family and transplantation

The third class comprises the smallest part of the classified corpus (13%). It can be divided into two subclasses.

“To bother + ”, “to recount”, “mother” and “colleague” belong to the “Talking about the illness” subclass. Patients sometimes choose (not) to speak about their illness. The quantity of details they go into depends on who they are speaking to. They do not want to overwhelm their relatives (Class 3, Excerpts 1–3).

Patients are also informers. “Hide”, “current” (found in the expression “au courant” which means know about/be informed), “world” (found in the expression “tout le monde”, literally “all the world”, everyone) and “hear” show with whom patients share information and their experience (Class 3, Excerpts 2–3).

The “Talking about transplantation” subclass includes “to give”, “compatible, “to decease” and “to refuse.” When patients think about transplantation, they also think about their family. Relatives may, for example, offer a kidney, which may or may not be possible (Class 3, Excerpt 4). Moreover, patients may refuse a donation perceived as unthinkable to accept (e.g., a son offering a kidney to his mother) (Class 3, Excerpt 5).

More rarely, patients mention they have asked relatives if they could be donors or have talked to them about deceased-donor transplantation (Class 3, Excerpts 6–7). Interestingly, “dialysis” is significantly absent from this class.

**Fig. 2:**
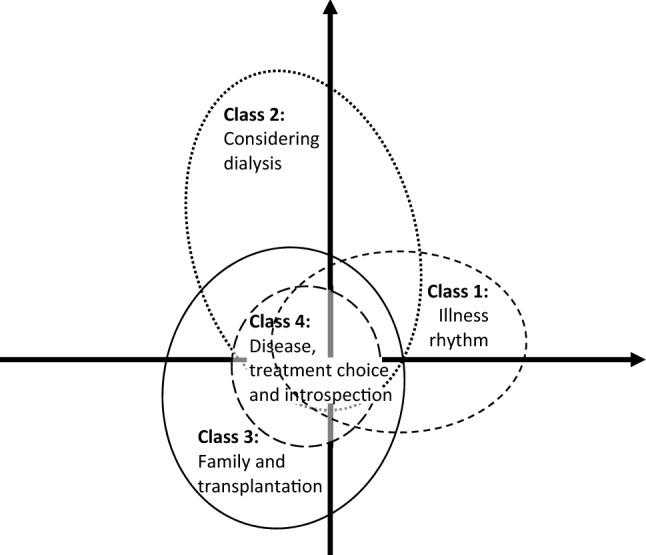
Schematic representation of the factorial analysis of patients’ discourse

Class 3 is associated with depression and attendance at patient education sessions. Moreover, Class 3 opposes Class 2 in the factorial correspondence analysis (Fig. 2). Dialysis (Class 2) and transplantation (Class 3) do not appear together in patients’ discourse. They belong to different decision-making processes.

#### Class 4: disease, treatment choice and introspection

The fourth class contains the largest portion of the classified corpus (43%). It includes markers of discursive relations (e.g., “but”, “if”) and modal words (e.g., “no”, “I think”). These terms show how this class pertains to reflections about CKD. It is divided into three subclasses.

The “A normal life” subclass highlights how patients say they have a “normal” “life.” Their illness is not an important element of their day-to-day life (e.g., “sick”, “health”).The only symptom they report is fatigue (Class 4, Excerpts 1–2).

However, some words temper this idea. A French expression equivalent to “quotation mark” (found in the expression “in quotation marks”), “impression”, “to evolve” and “to arrive/to happen” show how this normality is perceived as relative. Patients know their disease will progress and fear its development (Class 4, Excerpts 3–4). “Haha”, “Pff” and other interjections show the participants’ affective attitudes (Class 4, Excerpt 5).

The “Avoiding thinking about CKD” subclass shows that as CKD has little impact on patients’ day-to-day life, participants say they do not “think” much about their illness (Class 4, Excerpt 6). However, words such as “instant” (found in the expression “for the instant”, for right now), “mind” and “happen” temper this conclusion. Patients do not want to “stress” in advance, but they are aware of the temporary nature of their situation (Class 4, Excerpt 7). “Try” and “manage” are used to describe the management of CKD. They can refer to present and future ways to cope with it (Class 4, Excerpts 9–10).

Class 4 is characterized by demonstratives, relatives and indefinites (e.g., “other”, “what + ”) that designate without naming. Moreover, “thing” is strongly associated with this class and subclass. These words are used to talk about CKD, KRT or their physical/psychological consequences (Class 4, Excerpt 8).

The subclass “Research participation, treatment choice and acceptance” shows how patients accept their illness under certain conditions (e.g., “to continue”). They do not think of themselves as sick. Therefore, “to accept” refers mainly to KRT (Excerpts 11–12). “Question” can refer to questions patients ask others (e.g., their nephrologist) and questions they ask themselves (e.g., about KRT). Moreover, patients feel decision-making is not a current concern, which makes “answering” the interview questions hard (Class 4, Excerpt 13).

This class is associated with patients who are neither depressed nor anxious.

## Discussion

This study is one of the first to interview a diverse sample of patients about treatment decision-making before KRT and to investigate participants’ experience with moderate to advanced CKD. It shows that statistical text analysis is an interesting method as it allows the simultaneous investigation of *what* participants are talking about and *how* they are doing it. Our results show that CKD is manifested for patients not through symptoms but through its monitoring. They also emphasize how common cognitive avoidance about disease progression is in pre-KRT patients. Cognitive avoidance encompasses a variety of coping strategies aimed at escaping thoughts about undesirable situations, including denial of the disease [27]. It may be used by patients to reduce their anxiety regarding KRT decision-making. Although effective for reducing negative emotions in the short-term, avoidance probably leads to heightened negative affects (e.g., depression, anxiety) in the long term, as has been shown in other populations [28, 29]. Moreover, transplantation and dialysis belong to two distinct decision-making processes. Family plays an important role in transplantation choice by sharing opinions and by (not) offering a kidney. The novelty of this study is that patients were interviewed before KRT and thus it provides an accurate and contemporaneous understanding of patients’ decision-making process.

The analysis encompasses 68% of the entire corpus, which is satisfactory according to recommendations (> 50%) [30]. The most important class in terms of analyzed corpus quantity shows that patients report having a normal life (class 4). It is patients’ monitoring that attests to the existence of CKD (class 1). What makes it stressful is not its physical impact but its progression towards kidney failure. Previous studies showed similar results [31, 32]. Patients' current health status may lead to cognitive avoidance, which may delay decision-making [31, 33]. It might nonetheless be adaptive in the short term, especially at stage 3, if it does not prevent patients from engaging in healthy behaviors. Further research should investigate the frequency and impact of cognitive avoidance in advanced-stage CKD patients.

Regarding KRT decision-making, dialysis choice seems guided by patient education, whereas family has a central role in decision-making about transplantation, especially living donation. Interestingly, family does not influence the consideration of dialysis in this pre-decision stage, according to patients. Yet, other authors have emphasized how family influences the choice of home dialysis [34]. Two reasons might explain these conflicting results. First, most past studies are retrospective [6, 8, 34]. Thus, patients may have experienced retrospective bias and overestimated family influence. Second, nearly half of our participants were at stage 3 and might not yet have reached this decision-making process. Patients may contemplate dialysis only when transplantation is impossible, while most of them undergo dialysis before transplantation [35]. Some patients have a strong aversion toward dialysis [36]. Consequently, some may talk about dialysis with their relatives in a second phase.

Classes identified by our analysis were associated with categorical variables. Class 1 (“Rhythm of the illness”) does not include KRT decision-making. It is associated with patients with stage 3 or 5 CKD whereas class 2 (“Considering Dialysis”) is associated with stage 4 CKD. This may show that treatment choice occurs during stage 4. Patients in stage 3 may not feel concerned by KRT whereas stage 5 patients may have already made their choice. This result is consistent with the current guidelines for KRT decision-making [5]. Class 4 is associated with low levels of depression and anxiety, it is characterized by self-reflection linked to better mood [37–39]. Treatment choice may be hindered by mental health issues. Indeed, several studies showed that decision-making is affected by depression and anxiety [40].

Limitations in our study warrant mention. First, it is cross-sectional and what the patients said reflected what they were experiencing at the time of the interview. Some participants were not facing impending KRT decision-making. Treatment choice requires further research aimed at advanced-stage patients to assess changes in their discourse. Second, despite the strengths of statistical text analysis, it is based on word count [24]. Yet people may use different words to describe similar experiences. Some topics could be mentioned once and be meaningful without being statistically significant (e.g., thoughts about death). Moreover, due to the qualitative nature of this study and the use of purposive sampling, the results are not generalizable. Indeed, qualitative research usually does not aim for generalizability but rather for transferability [41]. Finally, we can hypothesize that the interviewees were more concerned by KRT as they are younger, more educated, with stage 4 or 5 CKD and anxious.

### Clinical perspectives

Investigating patients’ perspective on CKD and their care path allows to open new perspectives to improve care and patients’ quality of life. Attending education programs to select KRT is important, but does not seem to be sufficient [42]. Patients should be educated and empowered to achieve the health outcomes and life goals that are meaningful and important to them, through communication and education skills, patient resilience, strengthening social connections, and access to support [42, 43]. Social workers, fellow patients and psychologists should be included in the conception of such programs in order to help reduce patient cognitive avoidance and decisional conflict [44]. Our results also highlight the importance of including patients’ families in educational interventions, especially regarding transplantation, which have proved to be effective [45]. Moreover, coordinating patients’ care pathways, as recently implemented for those with CKD stage 4 or higher in France, could reduce cognitive avoidance and lead to more satisfying KRT choices.

## Conclusion

This study underlines that cognitive avoidance is common in patients and that dialysis and transplantation belong to two distinct decision-making processes. Although cognitive avoidance may be adaptive, healthcare teams should be watchful as it may affect patients’ well-being and their satisfaction with decision-making. Patients seem not to contemplate dialysis when transplantation is an option and may rush their dialysis modality choice when reaching kidney failure. Finally, family plays an important role in treatment choice, especially when transplantation is considered. Thus, caring for patients and their families seems relevant during KRT decision-making. Further research should investigate relatives’ experiences to better understand their role.

## Supplementary Information

Below is the link to the electronic supplementary material.

Supplementary file1 (PDF 209 KB)

Supplementary file2 (DOCX 14 KB)

Supplementary file3 (DOCX 28 KB)

Supplementary file4 (DOCX 15 KB)

Supplementary file5 (DOCX 13 KB)
