# Supplementary material for: Patient perspectives on chronic kidney disease and decision-making about treatment. Discourse of participants in the French CKD-REIN cohort study
Source: J Nephrol. 2022 Jun 13;35(5):1387–97. doi: 10.1007/s40620-022-01345-6 (PMC9217839; doi:10.1007/s40620-022-01345-6)
Supplement: Supplementary file 1 — Supplementary file1 (PDF 209 KB) [file 40620_2022_1345_MOESM1_ESM.pdf]

Supplementary material: Results of the Ascending Hierarchical Analysis

Class 1

- Done/Fact (Chi^2 = 29)
- Nephrologist (Chi^2 = 56)
- See (Chi^2 = 36)
- Do again (Chi^2 = 31)
- Generalis*/GP (Chi^2 = 29)
- Renal (Chi^2 = 39)
- Potassium (Chi^2 = 22)
- Creatine (Chi^2 = 22)
- Test/Taken/Sample (Chi^2 = 129)
- Blood (Chi^2 = 159)
- Analysis (Chi^2 = 105)
- Result (Chi^2 = 171)
- Exam (Chi^2 = 75)
- List (Chi^2 = 29)
- Discovered (Chi^2 = 21)
- Go up (Chi^2 = 63)
- Go down (Chi^2 = 24)
- Stable (Chi^2 = 42)
- Rate/Level (Chi^2 = 30)
- Clearance (Chi^2 = 42)
- Creatinine (Chi^2 = 50)
- Push (Chi^2 = 22)
- Cyst (Chi^2 = 27)
- To puncture (Chi^2 = 25)
- Monday (Chi^2 = 31)
- Number (Chi^2 = 33)
- Urgent (Chi^2 = 32)
- Time (Chi^2 = 23)
- Last (Chi^2 = 108)
- To control (Chi^2 = 22)
- April (Chi^2 = 27)
- To see again (Chi^2 = 28)
- Next (Chi^2 = 25)
- January (Chi^2 = 29)
- Month (Chi^2 = 335)
- Nephro+ (Chi^2 = 83)
- Appointment (Chi^2 = 57)
- Cardio+ (Chi^2 = 39)

CKD Monitoring

- Medica+ (Chi^2 = 35)
- Cardiologist (Chi^2 = 39)
- To follow/To be monitored (Chi^2 = 25)
- Checkup (Chi^2 = 50)
- Prescri+ (Chi^2 = 21)
- Gone (Chi^2 = 21)
- To treat (Chi^2 = 34)
- Physician (Chi^2 = 26)
- To send (Chi^2 = 40)
- Year (Chi^2 = 50)
- Infarction (Chi^2 = 21)
- Surge+ (Chi^2 = 35)
- Scanner (Chi^2 = 35)
- Urologist (Chi^2 = 30)
- To call (Chi^2 = 33)
- Doctor (Chi^2 = 50)
- [Name] (Chi^2 = 35)
- July (Chi^2 = 22)
- To carry (Chi^2 = 27)
- Pill (Chi^2 = 34)
- To get up/To raise (Chi^2 = 24)
- Morning (Chi^2 = 45)
- Insulin (Chi^2 = 22)
- Crisis/Episode (Chi^2 = 32)
- Low/Bottom (Chi^2 = 25)
- December (Chi^2 = 22)
- Put/Place (Chi^2 = 28)
- Pain (Chi^2 = 40)
- Watch/Monitor (Chi^2 = 32)
- Left (Chi^2 = 29)
- Finger (Chi^2 = 25)
- Aperçu (Chi^2 = 22)
- Tension/Pressure (Chi^2 = 92)
- To raise (Chi^2 = 25)
- Progress/Develop (Chi^2 = 26)
- Produce/Product (Chi^2 = 27)

Managing an illness among others

Class 2

- Dialys+ (Chi^2 = 355)
- Peritoneal+ (Chi^2 = 298)
- To do (Chi^2 = 15)
- Autonom+ (Chi^2 = 73)
- Hospital (Chi^2 = 155)
- Week (Chi^2 = 24)
- Hemodialysis (Chi^2 = 137)
- To know (Chi^2 = 17)
- Sort (Chi^2 = 45)
- To take/To pass/To go (Chi^2 = 14)
- House/Home (Chi^2 = 149)
- Possib+/Potential (Chi^2 = 17)
- Apparently (Chi^2 = 15)
- Way/Type (Chi^2 = 56)
- Chosen (Chi^2 = 17)
- Bedroom (Chi^2 = 93)
- Litre (Chi^2 = 22)
- Centre (Chi^2 = 94)
- Technique (Chi^2 = 13)
- Indeed (Chi^2 = 21)
- To constrain (Chi^2 = 35)
- Domicile/Home (Chi^2 = 137)
- Night (Chi^2 = 114)
- Free (Chi^2 = 28)
- Day (Chi^2 = 70)
- Hour (Chi^2 = 80)
- To sleep (Chi^2 = 33)
- To opt/To choose (Chi^2 = 44)
- To depend (Chi^2 = 40)
- To choose (Chi^2 = 30)

Dialysis modalities and daily life

- Method/Way (Chi^2 = 135)
- To show (Chi^2 = 50)
- As well (Chi^2 = 21)
- Tube/tubing/tip (Chi^2 = 73)
- Stomach/Belly (Chi^2 = 60)
- Catheter (Chi^2 = 25)
- Arm (Chi^2 = 109)
- Fistula (Chi^2 = 21)
- Constraint (Chi^2 = 104)
- Freedom (Chi^2 = 14)
- To displace/to move (Chi^2 = 49)
- Connect/plug (Chi^2 = 72)
- Machine (Chi^2 = 187)
- Device/Instrument (Chi^2 = 60)
- Pouch/Medical bags (Chi^2 = 30)
- Disabled (Chi^2 = 22)
- Functioning (Chi^2 = 31)
- Information (Chi^2 = 32)
- Meeting (Chi^2 = 14)
- Represent (Chi^2 = 33)
- To can/To be able (Chi^2 = 7)
- Different (Chi^2 = 42)
- Spot/place (Chi^2 = 28)
- To live/To reside (Chi^2 = 25)
- Village (Chi^2 = 28)
- To connect/to plug in (Chi^2 = 56)
- Place (Chi^2 = 23)
- Holidays (Chi^2 = 25)
- To enquire (Chi^2 = 27)
- System (Chi^2 = 23)
- Disadvantage (Chi^2 = 33)
- Advantage (Chi^2 = 38)

Patient education and treatment choice

Class 3

- Mother (Chi^2 = 126)
- To bother/Bother (Chi^2 = 34)
- Familial (Chi^2 = 13)
- To come back/To go into (Chi^2 = 22)
- To recount (Chi^2 = 71)
- Misfortune (Chi^2 = 26)
- Colleague (Chi^2 = 33)
- To receive (Chi^2 = 46)
- Contact+ (Chi^2 = 15)
- Sunday (Chi^2 = 40)
- Pal (Chi^2 = 47)
- Laugh (Chi^2 = 27)
- To laugh/to giggle/laughter (Chi^2 = 17)
- Niece (Chi^2 = 53)
- Nephew (Chi^2 = 33)
- Family (Chi^2 = 200)
- Parent (Chi^2 = 73)
- To talk (Chi^2 = 38)
- Sister (Chi^2 = 261)
- Eldest (Chi^2 = 40)
- Brother (Chi^2 = 352)
- Together (Chi^2 = 54)
- To hear (Chi^2 = 20)
- Kid (Chi^2 = 33)
- Mom (Chi^2 = 21)
- World (Chi^2 = 47)
- Friend (Chi^2 = 50)
- Entourage (Chi^2 = 30)
- To hide (Chi^2 = 17)
- Current (Chi^2 = 16)

Talking about the illness

- Donation (Chi^2 = 42)
- Research (Chi^2 = 15)
- Woman (Chi^2 = 19)
- Living/Alive (Chi^2 = 65)
- Bro (Chi^2 = 33)
- To give (Chi^2 = 68)
- Compatible (Chi^2 = 92)
- Bip (Chi^2 = 29)
- To refuse (Chi^2 = 17)
- Father (Chi^2 = 114)
- To die (Chi^2 = 71)
- Husband (Chi^2 = 133)
- Step sister/Sister in-law (Chi^2 = 29)
- Son (Chi^2 = 171)
- To take to (Chi^2 = 27)
- To handle/to sort out (Chi^2 = 17)
- Child (Chi^2 = 160)
- Here/there/at (Chi^2 = 68)
- Young (Chi^2 = 29)
- Girl/Daughter (Chi^2 = 162)
- Boy (Chi^2 = 80)
- Oxygen (Chi^2 = 33)
- Race/Errand (Chi^2 = 35)
- To drive (Chi^2 = 33)
- Dead (Chi^2 = 29)
- Group (Chi^2 = 19)
- Perturb+ (Chi^2 = 17)
- In-law/step/beautiful (Chi^2 = 53)

Talking about transplantation

Class 4

- Choice (Chi^2 = 19)
- Quotation marks (Chi^2 = 15)
- To evolve/to progress (Chi^2 = 17)
- Blow/shock/assault (Chi^2 =20)
- Haha (Chi^2 = 45)
- To say (Chi^2 = 52)
- Pff (Chi^2 = 15)
- Action (Chi^2 = 21)
- Life (Chi^2 = 24)
- Important (Chi^2 = 19)
- Impression (Chi^2 = 19)
- To feel (Chi^2 =13)
- Normal (Chi^2 =32)

A normal life

- Mind (Chi^2 = 16)
- State (Chi^2 = 17)
- Health (Chi^2 = 25)
- Negative (Chi^2 = 16)
- Subject (Chi^2 = 21)
- To stop (Chi^2 = 22)
- To manage (Chi^2 = 20)
- Psychologist (Chi^2 = 18)
- Moment (Chi^2 = 31)
- Live (Chi^2 = 37)
- Vivre (Chi^2 = 22)
- Sense (Chi^2 = 34)
- To arrive/to happen (Chi^2 = 14)
- Instant (Chi^2 = 33)
- Stress (Chi^2 = 15)
- To try (Chi^2 = 37)
- Thing (Chi^2 = 67)
- To think (Chi^2 = 43)

Avoiding thinking about CKD

- Sick (Chi^2 = 20)
- To accept (Chi^2 = 36)
- Head (Chi^2 = 15)
- True (Chi^2 = 14)
- Relationship (Chi^2 = 17)
- Decision (Chi^2 = 35)
- To answer (Chi^2 = 20)
- Question (Chi^2 = 35)
- To ask/To put (Chi^2 = 26)
- In the end (Chi^2 = 16)
- Opinion (Chi^2 = 14)
- Clear (Chi^2 = 18)
- To continue (Chi^2 = 20)
- Today (Chi^2 = 6)

Research participation, treatment choice and acceptance
