# Supplementary material for: Patient perspectives on chronic kidney disease and decision-making about treatment. Discourse of participants in the French CKD-REIN cohort study
Source: J Nephrol. 2022 Jun 13;35(5):1387–97. doi: 10.1007/s40620-022-01345-6 (PMC9217839; doi:10.1007/s40620-022-01345-6)
Supplement: Supplementary file 2 — Supplementary file2 (DOCX 14 KB) [file 40620_2022_1345_MOESM2_ESM.docx]

**Supplementary material:** Description of ALCESTE® analysis

ALCESTE® performs a lemmatization of the corpus: words with the same lexical roots are gathered under the same spelling indicated by a “+” (e.g., cardio+ can refer to “cardiologist” or “cardiology” etc.). Statistical analyses are then performed on the words used by participants, based on punctuation and significant word distribution. The corpus is split into units of context (UCs), roughly equivalent to sentences. ALCESTE® classifies the UCs into lexical classes. These classes are established with a descending hierarchical classification (DHC) accounting for words’ distribution and co-occurrence. Then, ALCESTE® performs a forward hierarchical classification (FHC) highlighting how words are associated with each other inside each class. This allows to identify subclasses. This type of analysis allows to identify which words appear conjointly with others. Each class is a cluster of words frequently pronounced in the same sentence or group of sentences by patients. It counts repetitions of associations of words sufficiently close together and thus enables to construct groups of words (i.e., classes) than can be interpreted as themes by the researchers. Conversely, “significant absences” are words that are negatively associated with a class. In other words, they are words that are not used at all with the other words composing the class. Grammatical words (e.g., prepositions), also called “supplementary forms”, are not considered in the *construction* of the classes by ALCESTE®, but they are used to describe each class they are associated with. A chi-square indicates the strength of the association between words and their class regardless if they are significantly present or absent. Moreover, ALCESTE® provides an output presenting the association of grammatical categories (e.g., demonstratives, nouns, numbers, words describing spatial relationships) with each class. It specifies that a Chi-square above 0 indicates a “relative presence” of this category in the class whereas a Chi-square equal to 0 means that the association between this category and the class is not significant. As a chi-square cannot be negative, when a minus precedes the Chi-square value it is used by the software to indicate a “relative absence” of the word in the class.

A Factorial Correspondence Analysis (FCA) is performed based on the results of the DHC. It allows to visualize the relationships between each class. A graphic representation of the FCA show the position of each class and word according to their link with the factors resulting from the analysis. Therefore, the researcher can characterize each factor by describing the correspondences and oppositions between classes regarding said factor (Bart, 2011).
