# Supplementary material for: Patient perspectives on chronic kidney disease and decision-making about treatment. Discourse of participants in the French CKD-REIN cohort study
Source: J Nephrol. 2022 Jun 13;35(5):1387–97. doi: 10.1007/s40620-022-01345-6 (PMC9217839; doi:10.1007/s40620-022-01345-6)
Supplement: Supplementary file 3 — Supplementary file3 (DOCX 28 KB) [file 40620_2022_1345_MOESM3_ESM.docx]

**Supplementary Material:** Characteristics of the sample and comparison between patients who consented to an interview and patients who did not

| **Variables** | **Whole sample**  N= 1555^1^ | **Patients who did not consent to an interview** | **Patients who consented to an interview** | **p** |
| --- | --- | --- | --- | --- |
|  |  | N = 786 | N = 769 |  |
| **Sociodemographics** |  |  |  |  |
| Age (Mean (SD)) | 69.5 (12.0) | 71.5 (11.2) | 67.4 (12.4) | <.001 |
| Gender (% women) | 33.5% | 32.3% | 34.7% | 0.34 |
| GFR (Mean (SD)) | 31.7 (13.5) | 31.4 (13.3) | 32.0 (13.7) | 0.37 |
| **Last known CKD stage** |  |  |  |  |
| Stage 2-3 | 50.7% | 50.0% | 51.5% | 0.59 |
| Stage 4-5 | 49.3% | 50.0% | 48.5% |  |
| **Attended patient education on KRT (% Yes)** | 15.4% | 13.0% | 17.9% | 0.02 |
| **Marital status** |  |  |  |  |
| Single | 10.4% | 9.6% | 11.5% | 0.004 |
| Divorced | 12.2% | 10.3% | 14.7% |  |
| Married | 63.6% | 66.0% | 63.8% |  |
| Widowed | 11.8% | 14.2% | 9.9% |  |
| NA | 2.0% | 2.0% | 2.0% |  |
| **Lives alone** | 23.2% | 23.4% | 23.0% | 0.73 |
| **Occupational situation** |  |  |  |  |
| Employed | 18.5% | 13.4% | 23.7% | <0.001 |
| Retired | 69.2% | 74.8% | 63.5% |  |
| Unemployed | 8.4% | 7.3% | 9.6% |  |
| NA | 3.9% | 4.6% | 3.3% |  |
| **Education level (years)** |  |  |  |  |
| ≤ 9 | 11.8% | 16.2% | 7.3% | <0.001 |
| 10 - 12 | 47.4% | 51.7% | 43.0% |  |
| > 12 | 39.7% | 30.9% | 48.8% |  |
| **Good literacy skills^1^** | 84.1% | 81.8% | 86.5% | 0.01 |
| **Depression^2^** |  |  |  |  |
| Mean (SD) | 7.0 (4.7) | 6.9 (4.6) | 7.1 (4.8) | 0.40 |
| Depressed | 36.1% | 34.1% | 38.1% | 0.41 |
| NA | 9.1% | 12.1% | 7.3% |  |
| **Anxiety^3^** |  |  |  |  |
| Mean (SD) | 5.6 (3.7) | 5.3 (3.6) | 5.9 (3.8) | 0.003 |
| Anxious | 24.2% | 21.0% | 27.4% | 0.01 |
| NA | 3.7% | 5.6% | 1.7% |  |
| **Discussion with family members** | 42.7% | 38.2% | 47.3% | 0.008 |
| NA | 5.7% | 8.4% | 2.9% |  |

GFR for glomerular filtration rate; NA for Missing Data; KRT for kidney replacement therapy; ^1^ Among the 1556 patients who returned the questionnaire, one was already undergoing dialysis and was thus excluded from further analyses; ^2^Measured with the Center of epidemiological studies depression scale (cut-off ≥ 8) (Kohout et al., 1993); ^3^Measured with the Hospital Anxiety and Depression Scale (cut-off ≥ 8) (Zigmond & Snaith, 1983)
