# Supplementary material for: Patient perspectives on chronic kidney disease and decision-making about treatment. Discourse of participants in the French CKD-REIN cohort study
Source: J Nephrol. 2022 Jun 13;35(5):1387–97. doi: 10.1007/s40620-022-01345-6 (PMC9217839; doi:10.1007/s40620-022-01345-6)
Supplement: Supplementary file 4 — Supplementary file4 (DOCX 15 KB) [file 40620_2022_1345_MOESM4_ESM.docx]

**Supplementary material:** Interpretation process

LM followed these steps in her interpretation process:

1. Familiarizing herself with the whole corpus *(e.g., interviews)*.
2. Familiarizing herself with all ALCESTE® outputs.
3. Reading the results of the Descending Hierarchical Classification (showing all the classes of the analysis and the significant presences and absences of lexical forms), the verbatims of each class, in particular the ones most associated with it. Determining a general idea of each class.
4. Carefully reading the associations between grammatical groups and each class. Refining the general idea identified at stage 3 and interpreting the linguistics aspects.
5. Identifying the subclasses with the FHC by cutting a perpendicular line through the schematic representation of the analysis. To determine the level of this perpendicular line, LM ensured it allowed to both summarize information and discriminate the subclasses, so they had a distinct meaning from one another.
6. Examining the subclasses identified at step 5 and the verbatims that are associated with the lexical forms that are characteristic of the subclasses. Interpreting the subclasses and refining the interpretation of the general class.
7. Examining the relationships between the classes with the factorial analysis. Interpret the axes of the FCA. Investigating the proximities, overlaps and oppositions between each class.
