# Supplementary material for: Patient perspectives on chronic kidney disease and decision-making about treatment. Discourse of participants in the French CKD-REIN cohort study
Source: J Nephrol. 2022 Jun 13;35(5):1387–97. doi: 10.1007/s40620-022-01345-6 (PMC9217839; doi:10.1007/s40620-022-01345-6)
Supplement: Supplementary file 5 — Supplementary file5 (DOCX 13 KB) [file 40620_2022_1345_MOESM5_ESM.docx]

**Supplementary material:** Subjectivity statement

| LM did a PhD in Health Psychology under the supervision of AU on the adaptation of patients living with CKD and their family members, with an interest in kidney replacement therapy decision-making and family influence on this process. This article presents results from her PhD thesis.  She has an interest in relationships, in particular family and those between patients and their healthcare providers.  She was introduced to statistical text analyses (including the Alceste method) by researchers in both psychology and linguistics. This explains her interest and focus on for grammatical markers in participants’ discourse. |
| --- |
